# Effect of food processing on the antioxidant activity of flavones from *Polygonatum odoratum* (Mill.) Druce

**DOI:** 10.1515/biol-2021-0010

**Published:** 2021-01-29

**Authors:** Guanghui Xia, Xinhua Li, Zhen Zhang, Yuhang Jiang

**Affiliations:** College of Food Science and Engineering, Tonghua Normal University, Tonghua 134001, China; College of Food Science, Shenyang Agricultural University, Shenyang 110866, China

**Keywords:** Polygonatum odoratum (Mill.) Druce, homoisoflavone, food processing methods, flavone antioxidant activity

## Abstract

*Polygonatum odoratum* (Mill.) Druce (POD) is a natural plant widely used for food and medicine, thanks to its rich content of a strong antioxidant agent called homoisoflavones. However, food processing methods could affect the stability of POD flavones, resulting in changes to their antioxidant activity. This study attempts to evaluate the antioxidant activity of POD flavones subject to different processing methods and determines which method could preserve the antioxidant activity of POD flavones. Therefore, flavones were extracted from POD samples, which had been treated separately with one of the four processing methods: extrusion, baking, high-pressure treatment, and yeast fermentation. After that, the antioxidant activity of the flavones was subject to *in vivo* tests in zebrafish embryos. The results show that yeast fermentation had the least disruption to the antioxidant activity of POD flavones, making it the most suitable food processing method for POD. By contrast, extrusion and high-pressure treatment both slightly weakened the antioxidant activity of the flavones and should be avoided in food processing. The research results provide a reference for the development and utilization of POD and the protection of its biological activity.

## 1 Introduction

*Polygonatum odoratum* (Mill.) Druce (POD) is a perennial herb belonging to the family Liliaceae. The root of POD serves both as a food source and a medicinal constituent. With a sweet and mild taste, POD root can act as a mucolytic agent in the lungs, mitigate intestinal problems [1], ease Yin deficiency-related dryness, and engender liquid to allay thirst [2].

There are various active substances in POD, namely, amino acids, polysaccharides, glycosides, and flavones [3,4]. Among them, flavones are antioxidants capable of slowing down aging, suppressing virus activity, curbing bacteria reproduction, and preventing cancers [5,6], as well as enhancing the immune system [7]. Moreover, flavones are regarded as a functional factor in healthy foods [8].

The antioxidant activity of flavones varies from material to material, owing to the difference in chemical structure. Therefore, some flavones could exhibit higher antioxidant activity than vitamin C or vitamin E [9,10]. The flavones in POD are mainly classified as homoisoflavones [11]. These homoisoflavones boast high antioxidant activity for the high content of phenolic hydroxyl groups. However, their antioxidant activity is affected by many factors, ranging from processing temperature, pressure to heating time. Any change in these factors could easily denature POD flavones, thereby changing their antioxidant activity.

Currently, POD is used as a raw material for many kinds of foods, such as bread, cake, wine, sauce, tea, and candy [12]. In recent years, POD has been used as a raw material for making rose cake, donkey-hide gelatin nutrition powder, and sheet jelly in China. These foods are produced by processing methods such as extrusion, fermentation, baking, and high-pressure treatment. Nevertheless, few scholars have explored how different processing methods affect the antioxidant activity of POD flavones. To improve POD food products' health function, it is necessary to identify the appropriate processing method that preserves POD flavones' antioxidant activity.

Zebrafish, a cypriniform teleost of the chordate phylum, share 87% of genes with human and possess similar biological structures and physiological functions to those of mammals [13]. The cellular signal transduction pathways of zebrafish bear a high resemblance with those of human, suggesting that the results of experiments on zebrafish help predict the feasibility of human trials. In 2003, the National Institute of Health (NIH) in the United States designated zebrafish as an important experimental animal. Since then, the NIH has recognized the toxicity and functions of compounds evaluated through zebrafish experiments [14]. Zebrafish have been widely used by researchers engaging in molecular biology, developmental research, cancer, obesity, infectious diseases, and environmental studies [15,16]. Additionally, the zebrafish model has become increasingly popular in the evaluation of pharmacological and functional foods. Nonetheless, no report evaluates the antioxidant activity of POD flavones through *in vivo* tests on *in vivo* zebrafish.

Based on the zebrafish model, this study evaluates the antioxidant activity of POD flavones subject to different processing methods and determines which method could preserve the antioxidant activity of POD flavones. The research results provide a reference for the development and utilization of POD and the protection of its biological activity.

## 2 Materials and methods

### 2.1 Materials and reagents

POD was purchased from Songjianghe Town, Baishan City, northeastern China’s Jilin Province. Methanol (chromatographic grade) was procured from Tedia Company, Inc., Farfield, Ohio, USA. Yeast was obtained from Angel Yeast Co., Ltd, Yichang, China. 2,2-Azobis(2-methylpropyl)dihydrochloride (AAPH) was acquired from Sigma-Aldrich, Missouri, USA. Sodium chloride, potassium chloride, calcium chloride, dimethyl sulphoxide (DMSO), alchlor (AlCl_3_), ethanol, glacial acetic acid, and magnesium sulphate, all of which are analytically pure, were bought from Shanghai Experiment Reagent Co., Ltd, Shanghai, China.

5,7,4ʹ-Trihydroxy-6-methyl dihydrohomoisoflavone was supplied by Shanghai Tongtian Biotechnology Co., Shanghai, China. 5,7,6ʹ-Trihydroxy-6,8-dimethyl-4ʹ-methoxy dihydrohomoisoflavone, 5,7,4ʹ-trihydroxy-6,8-dimethyl dihydrohomoisoflavone, 5,7,4ʹ-trihydroxy-6-methyl-8-methoxydihydrohomoisoflavone, and 5,7-dihydroxy-6-methyl-8,4ʹ-dimethoxy-dihydrohomoisoflavone were prepared by preparative high-performance liquid phase in our lab (structural appraisal was completed by Shanghai Tongtian Biotechnology Co.).

Zebrafish were purchased from the Institute of Evolution & Marine Biodiversity, Ocean University of China. Malondialdehyde (MDA), reactive oxygen species (ROS), and superoxide dismutase (SOD) testing kits were purchased from the Nanjing Jiancheng Bioengineering Institute, Nanjing, China.

### 2.2 Preparation of POD powder

POD was cleaned before being placed in an air drying oven to be dried at 70°C. The dried material was ground into powder and then filtered through a sieve with a pore size of 425 µm.

### 2.3 POD treatments

#### 2.3.1 Extrusion

POD powder was extruded in a three-stage temperature-controlled extruder, using a granular cutter head at the outlet. The first, second, and third stages were set to be 45, 70, and 105°C, respectively. Before extrusion, 5,000 g of POD powder was spread on the flat surface of the extruder and evenly sprayed with water till the water content reached about 5%. Then, the wet POD powder was extruded for 80 s in the extruder. After that, the extruded products were cooled to room temperature and then ground with the said sieve.

#### 2.3.2 Yeast fermentation

Yeast (50 g) was placed in distilled water (500 g) at 26°C along with 3 g sugar for activation. After 15 min, 5,000 g of POD powder was placed in a glass tank and the yeast activated aqueous solution and 3,500 g of distilled water was added. After mixing to a smooth dough, the tank was placed into a constant-temperature incubator for fermentation at 28°C for 6 h. After that, the dough was dried in a hot-blast stove at 70°C [17]. The dried dough was ground as previously mentioned.

#### 2.3.3 Baking

POD powder (5,000 g) was put into a basin and turned into a dough by adding 4,000 g of distilled water. The dough was spread onto a baking plate and placed in the oven to be dried at 200°C. The dried dough was cooled to room temperature and ground with a screener.

#### 2.3.4 High-pressure treatment

POD powder (5,000 g) was moistened with 4,000 g of distilled water and placed in a portable autoclave in batches. The high-pressure treatment lasted 15 min at 121°C. After that, the powder was dried in a hot-blast stove at 70°C. The dried powder was cooled to room temperature and ground as previously described.

### 2.4 Extraction and purification of flavones

The POD powder processed by each method was dissolved in 95% ethanol at the ratio of 1:10 (weight to volume), thoroughly mixed, and ultrasonically extracted for 20 min. Then it was filtered, the filtered residue was repeat extracted twice, and the filtrate was merged. Then, the filtrate was rotary-evaporated to volatile ethanol and the extractum was obtained. The extractum was diluted with distilled water two times and decolorized with the same volume of petroleum ether five times. The decolorized solution was added prior to filtration in treated macroporous resin D101 columns at a flow rate of 1 mL/min. Each sample was pumped into its corresponding column and kept on the resin for 2 h. Next, each column was washed with 30, 70, and 100% ethanol solution, respectively. After washing, the 70% ethanol solution was collected, and most of the ethanol was rotary-evaporated from the sample. The residual solution was freeze-dried in vacuum to obtain the sample powder of POD flavone.

### 2.5 Plotting of standard curves and determination of flavone content

**Figure 1:**
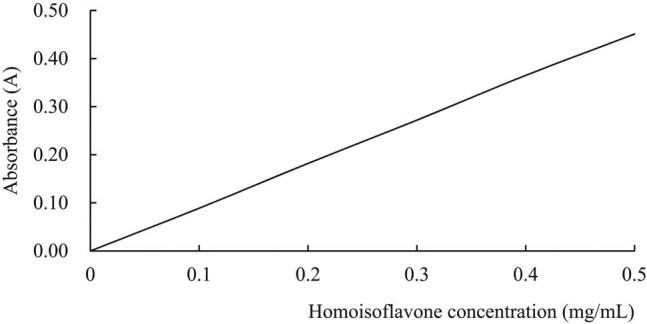
Homoisoflavone standard curve.

The standard of 5,7,4ʹ-trihydroxy-6-methyl dihydrohomoisoflavone was weighed to 100 mg and placed in a clean small beaker, before adding 50 mL of 50% chromatographic methanol solution. The mixture was subject to ultrasonic dissolution, and then transferred to a 100 mL volumetric flask. After cleaning solution was added to the volumetric flask, 50% chromatographic methanol solution was used to make the volume constant to produce a 1 mg/mL standard solution. Then, the standard curves of 0, 0.1, 0.2, 0.3, 0.4, 0.5, and 0.6 mg/mL were prepared with 1 mg/mL standard solutions. Each standard sample (1 mL) was added to a colorimetric tube with 1 mL of 1% AlCl_3_ solution, shaken well, and heated in a water bath at 45°C for 30 min. After that, the absorbance of each sample was determined at 415 nm with a spectrophotometer [18,19]. Taking absorbance (A) as the ordinate and concentration (C) as the abscess abscissa, a standard curve was drawn (as shown in Figure 1). The measured absorbance was substituted in the standard equation *y* = 0.9066 × −0.0001 (correlation coefficient, *R* ^2^ = 0.9999) to obtain the flavone content. Since the higher the flavone concentration is, the greater the absorbance value is, the total flavonoid concentration in the sample can be calculated according to the standard curve.

### 2.6 Zebrafish feeding and embryo collection

Zebrafish were raised and fed brine shrimp in a special breeding tank under the following conditions described in Westerfield [20]: temperature = 26–30°C; ventilation = 10 times/h; light/dark cycles = 14 h (light)/10 h (dark); daylight intensity ≥150 lux; pH of water = 6.8–7.2; dissolved oxygen = 10 mg/L; electrical conductivity = 500 S; water replacement: 10% per day; and feeding frequency = twice per day (once in the morning and once at night).

Healthy adult fish, half male and half female, were raised for more than 3 weeks, before being relocated to mating tanks and then artificial incubators. The temperature was controlled at 28°C, and feeding was stopped for 1 day. The barrier between the mating tanks was removed during light hours, allowing the males and females to mate and lay eggs. Once laid, the eggs fell to the bottom of the cylinder and were immediately collected, put into a clean petri dish, and cleaned with a culture solution. After the impurities were removed, the eggs were added to the culture solution at 28°C under constant temperature. Around 4 h post fertilization, the dead eggs were removed, and the remaining eggs were washed again and allowed to continue culturing.

The culture solution was prepared by the following mix ratio: 10% sodium chloride, 0.3% potassium chloride, 0.3% calcium chloride, and 0.8% magnesium sulphate in ultrapure water.


**Ethical approval:** The research related to animal use has been complied with all the relevant national regulations and institutional policies for the care and use of animals.

### 2.7 Measurement of antioxidant activity of POD flavones

Studies have shown that the 4 mg/mL AAPH could induce an oxidative stress reaction in zebrafish embryos [21,22,23]. The reaction produces an excessive amount of ROS, which damages embryo cells. The cell damage is typically represented by the surging MDA and SOD contents. If unable to clear the soaring ROS levels, an embryo will die.

To measure the antioxidant capacity of POD flavones, an appropriate concentration of POD flavones can inhibit the production of ROS and keep the MDA and SOD in the embryo at normal levels. The antioxidant activity of POD flavones is negatively correlated with its concentration required to maintain the normal levels of MDA and SOD.

Flavones of four different concentrations (10, 20, 50, and 100 μg/mL) were prepared from the processed samples by the culture solution (it was confirmed that the 200 µg/mL solution was toxic to zebrafish embryo). To improve the solubility of POD flavones, 0.001% DMSO was added in each solution preparation (this concentration has been proved nontoxic to zebrafish embryos).

The experiments were divided into fermentation group (PDCO-F), high-pressure treatment group (PDCO-H), extrusion group (PDCO-E), baking group (PDCO-B), nontreatment group (PDCO-N), and a blank control group. During the experiments, three parallel tests were conducted for sample of each concentration in each test group, and 2 mL of sample solution was successively added into the 24-well culture plate. In the blank control group, 2 mL of culture solution containing 0.001% DMSO was added. Six normally developed zebrafish embryos 8 h post fertilization were added to each of the sample-containing wells. Then, the culture plate was quickly transferred to a constant-temperature incubator at 28°C. An hour later, 2 mL of 8 mg/mL AAPH solution was added to each well to induce oxidative stress. After incubation for 3 h, the survival rates and normal development rates of the embryos were counted, and the ROS content, MDA activity, and SOD activity were measured to evaluate the antioxidant effect.

### 2.8 Calculation of ROS content, MDA activity, and SOD activity

#### 2.8.1 Calculation of ROS content

After the zebrafish embryos had been cultured, the embryos were filtered out and washed with normal saline. Each embryo was then homogenized with a tissue homogenizer, producing 10% tissue homogenate. Then, 1 mmol/L 2ʹ,7ʹ-dichlorodihydrofluorescein diacetate (DCFH-DA) working solution and phosphate-buffered saline (PBS) solution were prepared.

Following the instructions of the ROS test kit, a 48-well plate was prepared by adding 380 μL of tissue homogenate and 20 μL of 1 mmol/L DCFH-DA working solution into the sample wells. Meanwhile, 380 μL of tissue homogenate and 20 μL of PBS solution were added to the control wells. After mixing, the plate was placed in a constant-temperature incubator at 37°C for 1 h incubation in darkness. Then, the absorbance was measured at 485 and 525 nm with a microplate reader to derive the ROS content by: ROS content (U/mL) = fluorescence intensity/interstitial fluid volume.

#### 2.8.2 Calculation of MDA activity

The 10% tissue homogenate obtained from zebrafish embryos in Section 2.8.1 was centrifuged at 4,000 rpm for 10 min, and the supernatant (S solution) was obtained. Three 10 mL centrifuge tubes were taken and labelled as sample tube, standard tube, and blank control tube, respectively. The sample tube was added with 0.2 mL of S solution, the standard tube with 0.2 mL of 10 nmol/L tetraethoxypropane solution, and the blank control tube with 0.2 mL of anhydrous ethanol. Then, 0.2 mL of solution A, 3 mL of solution B, and 1 mL of solution C were added into the sample tube, standard tube, and blank control tube, respectively. Each centrifuge tube was well mixed with a vortex mixer and kept in a water bath at 95°C for 40 min. After rapid cooling with running water, the tubes were centrifuged at 4,000 rpm for 10 min, and the supernatant was collected to determine the absorbance of each tube at 532 nm (solution A, solution B, and solution C were all special solutions in MDA test kit):

\begin{array}{l}\text{MDA}\hspace{.25em}\text{content}\hspace{.25em}(\text{nmol/L})=100\times (\text{OD}\hspace{.25em}\text{value}\hspace{.25em}\text{of}\hspace{.25em}\text{sample}\\ \hspace{1em}-\text{OD}\hspace{.25em}\tex{value}\hspace{.25em}\text{of}\hspace{.25em}\text{blank}\hspace{.25em}\text{control})/(\text{OD}\hspace{.25em}\text{value}\hspace{.25em}\text{of}\hspace{.25em}\text{standard}\\ \hspace{1em}-\text{OD}\hspace{.25em}\text{value}\hspace{.25em}\text{of}\hspace{.25em}\text{blank}\hspace{.25em}\text{control}).\end{array}

#### 2.8.3 Calculation of SOD activity

Two 5 mL centrifuge tubes were taken and labelled as a sample tube and a blank control tube, respectively. The sample tube and the blank control tube were added with 1 mL of solution D. Then, the sample tube was added with 0.05 mL of solution S, while the blank control tube with 0.05 mL of distilled water. Afterwards, 0.1 mL of solution E, 0.1 mL of solution F, and 0.1 mL of solution G were added into the sample tube and the blank control tube, respectively. The centrifuge tubes were well mixed with a vortex mixer and kept in a water bath at 37°C for 40 min. Subsequently, to each tube 2 mL of chromogenic agent solution H was added. After standing for 10 min, the absorbance of each tube was measured at 550 nm (solutions D, E, F, G, and H were all special solutions in the SOD test kit):

\begin{array}{l}\text{SOD}\hspace{.25em}\text{activity}\hspace{.25em}\text{of}\hspace{.25em}\text{sample}\hspace{.25em}(\text{U/mL})=0.5\\ \hspace{1em}\times (\text{total}\hspace{.25em}\text{volume}\hspace{.25em}\text{of}\hspace{.25em}\text{reaction}\hspace{.25em}\text{solution/sampling}\hspace{.25em}\text{volume})\\ \hspace{1em}\times \text{dilution}\hspace{.25em}\text{ratio}\hspace{.25em}\text{of}\hspace{.25em}\text{sample}\times (\text{OD}\hspace{.25em}\text{value}\hspace{.25em}\text{of}\hspace{.25em}\text{control}\\ \hspace{1em}-\text{measured}\hspace{.25em}\text{OD}\hspace{.25em}\text{value})\text{/OD}\hspace{.25em}\text{value}\hspace{.25em}\text{of}\hspace{.25em}\text{control}\text{.}\end{array}

### 2.9 Qualitative analysis of extracted POD flavones with HPLC

The concentrations of the flavones in the treated POD flavone samples were determined by high-performance liquid chromatography (HPLC) on a 4.6 mm × 250 mm (5 μm) YMC-Pack ODS-AM column using an Agilent G7111A pump and a UV detector at 297 nm. The mobile phase consisted of methanol and 0.1% glacial acetic acid solution (60/40, v/v) with a flow rate of 0.8 mL min^−1^. The treated POD flavone samples (not lyophilized) were diluted 100 times with 60% methanol and were filtered prior to HPLC analysis. The injection volume and column temperature were 20 μL and 25°C, respectively.

## 3 Results and discussion

### 3.1 Flavone contents of the samples treated by different methods

The total flavonoid contents of POD flavone samples treated by different methods were determined (Table 1) after the samples were purified by D101 macroporous resin.

**Table 1:** Total flavonoid content of samples treated by different methods

Sample name	Sample mass (mg)	Solution volume (mL)	Absorbance (A)	Flavone concentration (mg/mL)	Total flavones content (%)
Nontreatment sample (control sample)	5	10	0.375	0.414	82.8
High-pressure treatment sample	5	10	0.344	0.379	75.8
Extrusion sample	5	10	0.343	0.378	78.21
Baking sample	5	10	0.303	0.334	75.6
Yeast fermentation sample	5	10	0.353	0.389	77.8

Table 1 shows that, after passing through D101 macroporous resin, the flavones were the main components in each sample, for the total flavone content surpassed 75% in every sample. This means flavones are an important factor affecting the results of the subsequent antioxidant tests.

### 3.2 Results on the antioxidant activity of POD flavones after different treatments

#### 3.2.1 Effect of POD flavones on embryo survival rate and malformation rate

**Figure 2:**
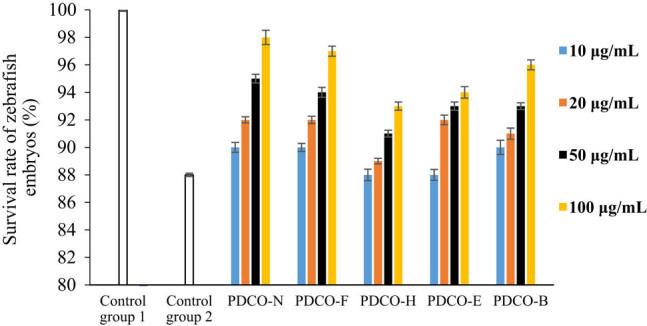
The effect of POD flavones on survival rate of zebrafish embryos. Note: zebrafish embryos were cultured in flavone solutions, in which the flavones were extracted from POD samples treated by different methods, or in control solutions, followed by a comparison between the effects of incubation in the various culture solutions on the survival rate. Control Group 1: culture solution + 0.001% DMSO; Control Group 2: culture solution + AAPH; PDCO-N: untreated POD flavone solution + AAPH; PDCO-F: yeast fermented POD flavone solution + AAPH; PDCO-H: high-pressure treated POD flavone solution + AAPH; PDCO-E: extruded POD flavone solution + AAPH; PDCO-B: baked POD flavone + AAPH.

**Figure 3:**
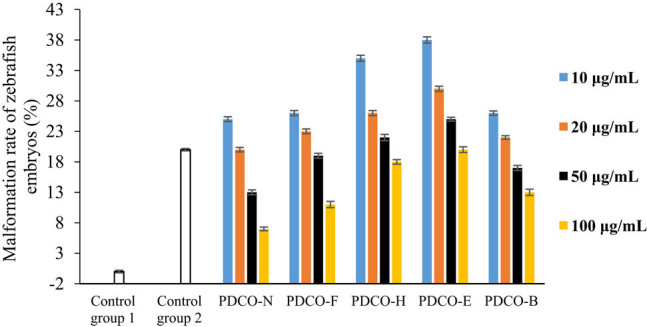
The effect of POD flavones on malformation rate of zebrafish embryos. Note: zebrafish embryos were cultured in flavone solutions, in which the flavones were extracted from POD samples treated by different methods, or in control solutions, followed by a comparison between the effects of incubation in the various culture solutions on the malformation rate. Control Group 1: culture solution + 0.001% DMSO; Control Group 2: culture solution + AAPH; PDCO-N: untreated POD flavone solution + AAPH; PDCO-F: yeast fermented POD flavone solution + AAPH; PDCO-H: high-pressure treated POD flavone solution + AAPH; PDCO-E: extruded POD flavone solution + AAPH; PDCO-B: baked POD flavone + AAPH.

As shown in Figures 2 and 3, zebrafish embryos grew regularly in Control Group 1 after 3 h of culture (survival rate: 100%; malformation rate: 0). In Control Group 2, however, malformations and deaths were observed, as evidenced by large yolk cysts and abnormal body segmentation. These phenomena resulted from the oxidative stress induced by 4 mg/mL AAPH. The survival rate was 88%, and the malformation rate was as high as 20% of cultured embryos.

Any concentration of POD flavones could mitigate the oxidative stress reaction of zebrafish embryos to AAPH. The greater the flavone concentration, the higher the survival rate, and the lower the malformation rate. The inhibition of oxidative stress was most effective when flavone concentration reached 100 μg/mL. It can also be observed from Figure 3 that both the untreated POD flavone sample in the control group and four kinds of processed POD flavone samples can lead to an increase in the malformation rate of zebrafish embryos at low concentrations, which may be caused by the impurities in the samples (referring to Table 1, there are still some impurities although the main component of each sample is flavone). These impurities may be new compounds formed during processing or reagent residues left in the extraction of flavone. In addition, the four kinds of processed POD flavone samples exert less impact on the survival rate of zebrafish embryos, indicating that the cytotoxicity of impurities in the samples is relatively lower; the cytotoxicity of impurities can be completely inhibited along with the increase in flavone concentration.

However, the embryos cultured in processed POD flavone solutions had a lower survival rate and a higher malformation rate than those in the control group (PDCO-N), which contains untreated flavone solution. Among the four groups of processed flavone solutions, the yeast fermented flavones (PDCO-F) were the most effective at improving survival rate. In PDCO-F, the malformation rate decreased with the flavone concentration.

#### 3.2.2 Effect of POD flavones on ROS content, SOD activity, and MDA activity

The ROS content in the untreated zebrafish embryos was about 4.2 × 10^5^ U/mL. Following AAPH induction, the ROS content reached 7.56 × 10^5^ U/mL (the blank control), about twice the normal level. This means 4 mg/mL AAPH successfully induced oxidative stress reaction. The higher the ROS content, the greater the damage to the embryos [24].

**Figure 4:**
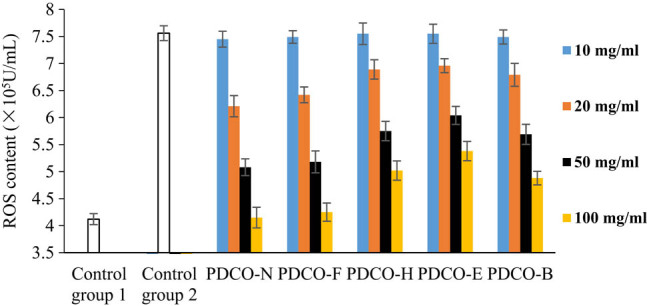
The effect of POD flavones on ROS content.

As shown in Figure 4, the POD flavone solutions treated by all four methods could reduce the ROS content in zebrafish embryos. The decrement of ROS content increased with flavone concentration. At each flavone concentration, PDCO-F boasted the most significant ROS suppression effect. When the flavone concentration reached 100 μg/mL, the ROS content in the embryos was slightly above that in the control group PDCO-N, but much lower than that induced by AAPH in Control Group 2. Hence, the POD flavones after yeast fermentation have a strong inhibitory effect on ROS.

PDCO-B, in which the flavones were extracted after baking, ranked the second in ROS inhibition. PDCO-E and PDCO-H were weaker in ROS inhibition than PDCO-F and PDCO-B, even as the concentrations of all flavones were increased. Compared with PDCO-E, PDCO-H showed a relatively strong inhibitory effect, but weaker than that of PDCO-B. The four groups of treated POD flavones can be ranked as PDCO-F, PDCO-B, PDCO-H, and PDCO-E in the descending order of ROS inhibition.

SOD, which naturally exists in human and other animals, is a beneficial enzyme that eliminates harmful O_2_^−^ produced during normal metabolism. As oxidative stress causes growing damage, SOD activity will be stimulated and continue to increase [25]. Thus, the abnormal increase of SOD activity helps identify whether the body is under increased oxidative stress.

**Figure 5:**
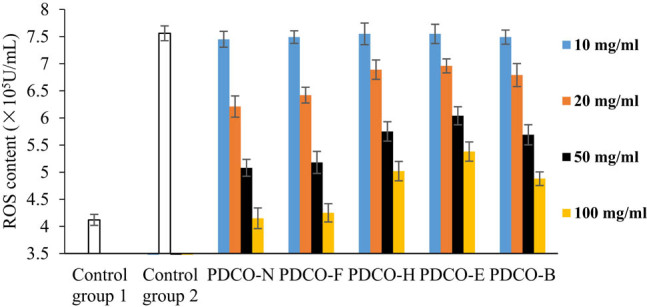
The effect of POD flavones on SOD activity.

As shown in Figure 5, after AAPH induced oxidative stress reaction, the SOD activity in zebrafish embryos surpassed 3.5 U/mL, far higher than that in the untreated control group (about 1 U/mL). The SOD activity plunged deeply after the use of POD flavones. The higher the flavone concentration, the sharper the decline of SOD activity.

In group PDCO-N, the SOD activity reached the normal level at the flavone concentration of 100 μg/mL. In group PDCO-F, the SOD activity approached the normal level also at the flavone concentration of 100 μg/mL. In group PDCO-E, however, POD flavones demonstrated a weaker effect on inhibition of the abnormal increase of SOD activity (*P* < 0.05).

**Figure 6:**
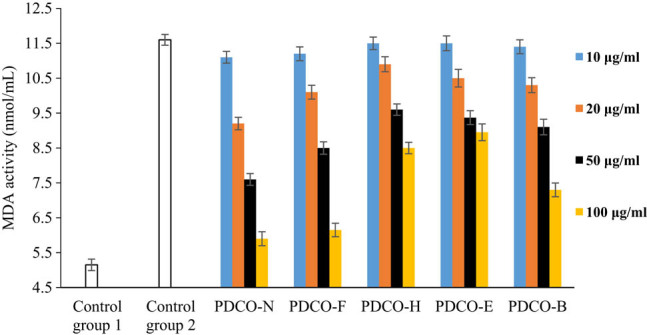
The effect of POD flavones on MDA activity.

MDA is an end product of membrane lipid peroxidation *in vivo*. The activity of MDA is an indicator of the severity of cell stress. A high MDA activity often indicates serious cell damage [25]. As shown in Figure 6, the MDA activity in the zebrafish embryos after AAPH induction was over two-fold higher that in the untreated control group, and the treatments with POD flavones significantly reduced the MDA activity in the embryos. The reduction is positively correlated with the flavone concentration.

In each of the four POD flavone solutions, the inhibitory effect on MDA activity increased with the flavone concentration. PDCO-F stood out for the strongest inhibitory effect. At each concentration, the inhibitory effect of group PDCO-F was only slightly behind that of control group PDCO-N. Despite being significantly lower than that of PDCO-F, the inhibitory effect of PDCO-B was more prominent than that of PDCO-H and PDCO-E. In PDCO-E, the inhibitory effect on MDA activity was poor, even when the flavones were of high concentration. The MDA activity in zebrafish embryos of this group was as high as 9 mmol/mL, far exceeding that in the untreated control group.

To sum up, the inhibitory effect of POD flavones on the oxidative stress reaction of zebrafish embryos, which mirrors the strength of antioxidant activity, depends on flavone concentration and treatment method.

#### 3.2.3 Analysis of antioxidant activity of POD flavones treated by different methods

**Figure 7:**
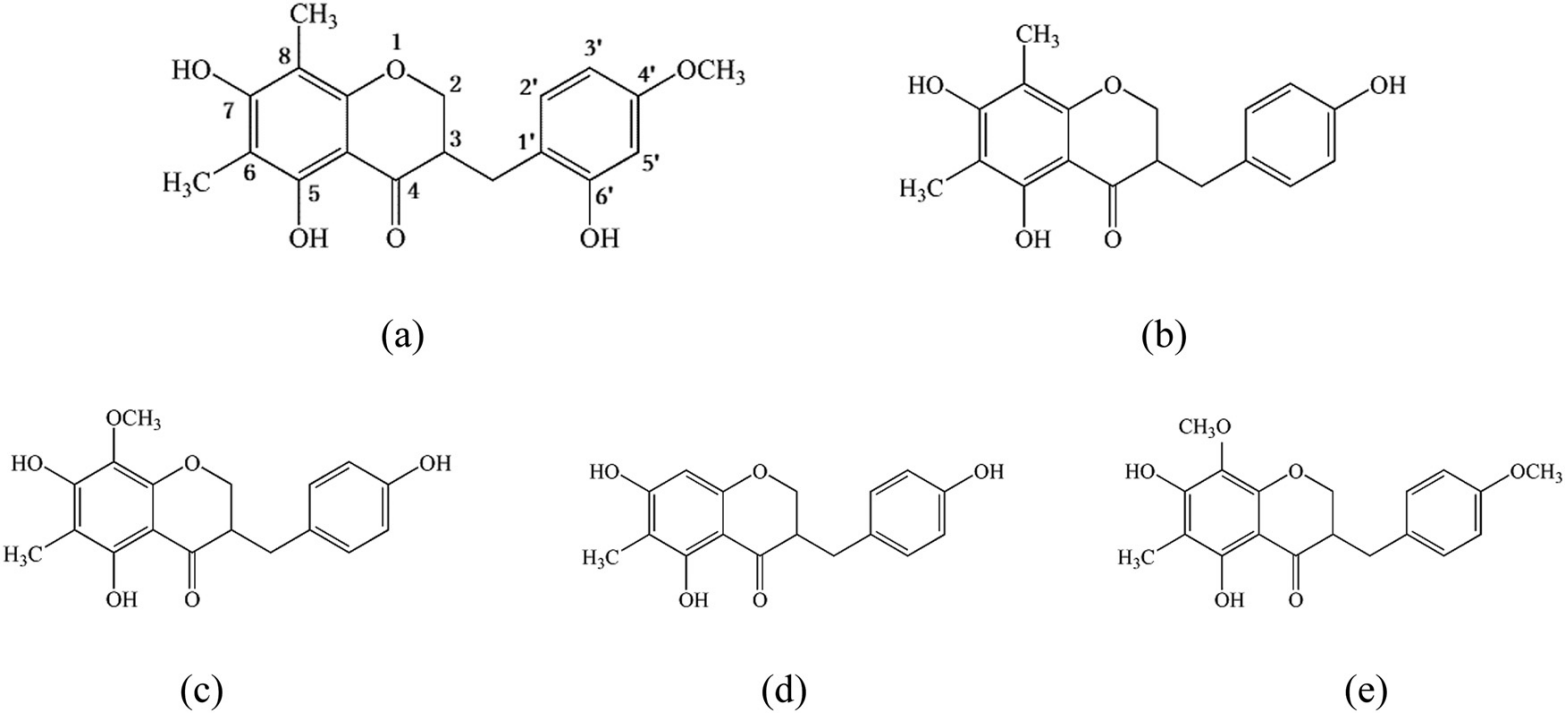
The molecular structure of five POD homoisoflavones. Note: (a) 5,7,6ʹ-trihydroxy-6,8-dimethyl-4ʹ-methoxy dihydrohomoisoflavone (POFa); (b) 5,7,4ʹ-trihydroxy-6,8-dimethyl dihydrohomoisoflavone (POFb); (c) 5,7,4ʹ-trihydroxy-6-methyl-8-methoxydihydrohomoisoflavone (POFc); (d) 5,7,4ʹ-trihydroxy-6-methyl dihydrohomoisoflavone (POFd); and (e) 5,7-dihydroxy-6-methyl-8,4ʹ-dimethoxy-dihydrohomoisoflavone (POFe).

In the aforementioned experiments, the POD flavones treated by different methods demonstrated different antioxidant activities. To disclose the mechanism behind the difference, five different homoisoflavone standard materials were taken as references. HPLC was used to detect the changes to the flavones in the treated POD flavone samples. The molecular structures of the five POD homoisoflavones are shown in Figure 7.

**Figure 8:**
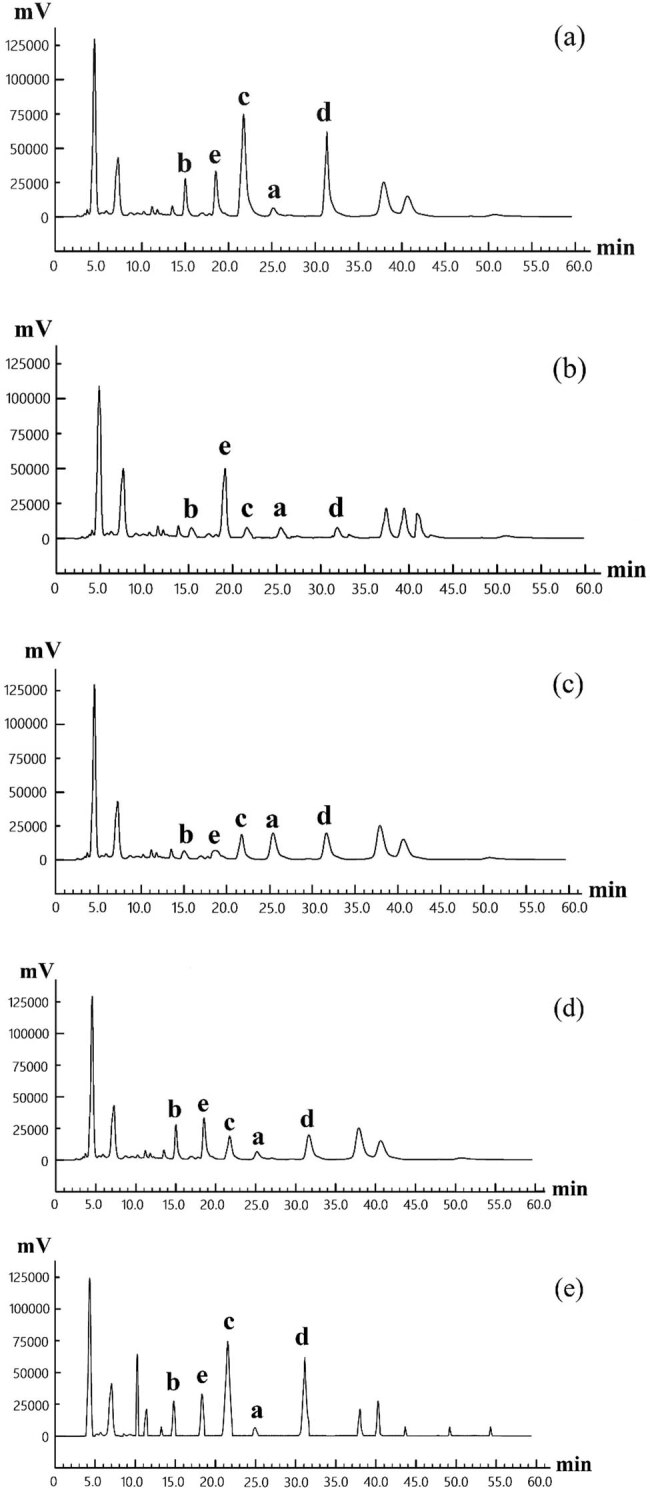
The HPLC chromatography of total flavones in each POD sample. Note: (a) yeast fermented sample; (b) high-pressure treated sample; (c) extruded sample; (d) baked sample; and (e) untreated sample.

The yeast fermented, high-pressure treated, baked, extruded, and untreated samples were analysed by HPLC. Then, the chromatogram of each sample is shown in Figure 8.

As shown in Figure 8, the yeast fermented sample, like the untreated control sample, contained a small amount and number of the five flavones. After high-pressure treatment, POFd, POFb, and POFc contents in total flavones of POD powder decreased (low concentration led to difficulty in detection), while POFe content increased. After extrusion, the contents of all five flavones exhibited a decrease. Cardoso et al. [26] also found that the contents of flavanones and flavones decreased sharply after extrusion cooking. After baking, the contents of POFd, POFb, and POFc in POD powder were on the decline.

Therefore, the variety and content of POD flavones changed following high-pressure treatment, extrusion, and baking, which may account for the change of antioxidant activity. A total of 11 flavones have been reported in the literature [27]. The antioxidant activity of these flavones could change with the positions and quantities of functional groups (e.g., phenolic hydroxyl groups). In addition, the stability of flavone molecules is affected by treatment conditions. Under high pressure or changing pressure, flavone molecules might crack or react with some components in food, bringing changes to antioxidant activity. Apart from the five homoisoflavones selected here, the other POD flavones identified in the literature may denature during food processing, and thus, alter the antioxidant activity of total flavones. This area requires further investigation.

How to avoid harmful denaturation is of great importance to preserve the antioxidant activity of POD flavones. Our research reveals that the antioxidant activity of POD flavone samples treated by yeast fermentation remained relatively unchanged. The result is consistent with that of Ravisankar et al. [28] since the authors found that the sourdough fermentation process commonly employed in rye bread processing did not negatively affect the flavone profile. Therefore, yeast fermentation is recommended for the production of POD foods.

## 4 Conclusions

This study carries out flavone extraction, purification, and antioxidant activity tests on the POD powder processed by baking, high-pressure treatment, extrusion, or yeast fermentation. The antioxidant tests on zebrafish embryos show that the POD flavones, treated by the four different methods, improved the survival rate of embryos after AAPH induced oxidative stress, reduced the malformation rate, lowered ROS content, and normalized MDA activity and SOD activity. Therefore, the flavones in POD powder treated by all four methods all maintained some antioxidant activity, which can protect body cells from or reduce oxidative stress damage. However, the treatment method brought changes to the type and content of flavones, which in turn altered antioxidant activity.

Compared with the untreated control group, the treated POD flavones had relatively low antioxidant activity. The least decrease in antioxidant activity was observed in the POD flavones treated by yeast fermentation, and the greatest decrease in those treated by extrusion.

The POD flavones treated by yeast fermentation maintained the strongest inhibitory effect on the oxidative stress reaction of zebrafish embryos, which mirrors the strength of antioxidant activity, followed by those treated by baking, those treated by high-pressure treatment, and those treated by extrusion.

To preserve the antioxidant activity of POD flavones, extrusion and high-pressure treatment are not recommended for food processing. The optimal treatment method is yeast fermentation; suitable products include POD bread, POD nutrition powder after fermentation, sheet jelly made of POD powder after fermentation, and so on.
